# Dramatic improvement of myotonia permanens with flecainide: a two-case report of a possible bench-to-bedside pharmacogenetics strategy

**DOI:** 10.1007/s00228-012-1414-3

**Published:** 2012-10-03

**Authors:** Jean-François Desaphy, Anna Modoni, Mauro LoMonaco, Diana Conte Camerino

**Affiliations:** 1 Section of Pharmacology, Department of Pharmacy, University of Bari - Aldo Moro, Bari, Italy; 2 Department of Neuroscience, Catholic University, Rome, Italy; 3 Dipartimento di Farmacia, Università degli Studi di Bari “Aldo Moro”, via Orabona 4 - campus, 70125 Bari, Italy

Sodium channel myotonias are inherited muscle diseases linked to mutations in the voltage-gated sodium (Nav1.4) channel [1]. Today, mexiletine is empirically considered as a first choice drug in non-dystrophic myotonias. Mexiletine likely acts by reducing muscle cell excitability through use-dependent Nav1.4 channel block. Nevertheless, not all patients benefit from mexiletine because of side effects (gastrointestinal discomfort, dizziness) or lack of efficacy.

Here we report the case of a mother and her son, both carrying the G1306E sodium channel mutation associated with a severe phenotype of myotonia permanens and little improvement with mexiletine. Since the G1306E mutant was more sensitive to flecainide than mexiletine in vitro [2], we decided to shift treatment to flecainide.

The proband came to our observation when she was 20 years old, complaining since early childhood of severe and painful generalized muscle stiffness, exacerbated after exercise, in wet weather and during febrile episodes. Generalized muscle hypertrophy was present. The lid-lag phenomenon lasted for several seconds, while relaxation time of the grip was lengthened to 30–40 s. Long exercise and cooling tests did not induce weakness. Electromyography showed normal nerve conduction but continuous myotonic running at rest. Serum creatine kinase was elevated to 400–900 IU/L. A biopsy of the left biceps muscle showed abnormal central nuclei and fiber size. The G1306E mutation was found in the SCN4A gene [3]. Previous electrophysiological studies described the alteration of mutant sodium channel gating and demonstrated its responsibility for the phenotype [3, 4]. During her life, the patient was given empiric trials of carbamazepine (3 × 200 mg/day), hydroquinidine (3 × 200 mg/day), tocainide (3 × 400 mg/day), and mexiletine (4 × 200 mg/day) with limited benefit.

During pregnancy (age 36 and 39 years), the proband experienced a striking worsening of myotonia. One son is affected, carrying the same G1306E mutation. He shows a very severe phenotype, including facial and ocular muscle myotonia since his birth, generalized painful muscle myotonia, and muscle hypertrophy. His general motor and mental development is normal. Since early childhood he has taken mexiletine with very poor benefit. Examination at age 5 years revealed forced internal rotation of the gleno-humeral joint and elevation of the shoulders, probably due to shoulder muscle stiffness and hypertrophy. Prolonged lid-lag phenomenon was found associated with severe orbicular myotonia, extraocular muscles and tongue myotonia.

On the basis of in vitro experiments [2], we decided to initiate treatment with flecainide. In the proband (age 43 years, body weight 65 kg), mexiletine (4 × 200 mg/day) was suspended in the afternoon and flecainide (100 mg/day) assumed next morning. Ambulatory cardiac function was evaluated. After 2 days of malaise, the patient started to improve. Four days later, flecainide dose was increased to 100 mg twice a day. The patient noticed an enhanced capability to move spontaneously after rest and a dramatic improvement of her periorbital and extraocular myotonia.

One month later, the proband child (age 7 years, body weight 25 kg) was changed from mexiletine to 35 mg flecainide twice a day. After 10 days, muscle stiffness decreased so that the internal rotation of the gleno-humeral joint and elevation of the shoulders were significantly reduced. The mother reported that he became able to have a meal and dress himself without any help. At last follow-up the child was taking 60 mg flecainide twice a day.

**Fig. 1:**
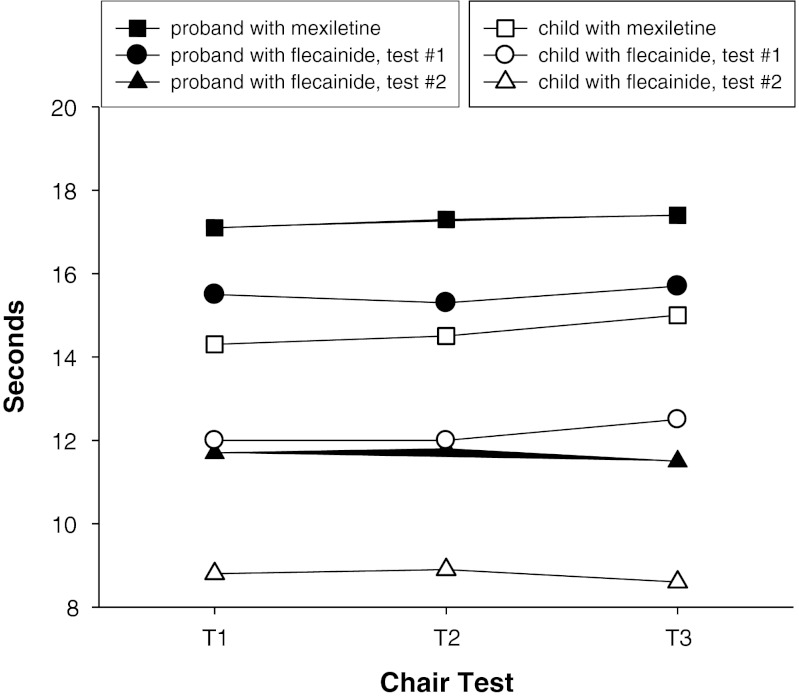
Chair test. The functional chair test was performed at the same time of day, in a comfortable room with an ambient temperature of 25 °C. Both patients were asked to rise from a chair after a 5 min rest and perform three turns around the chair as quickly as they could (T1). The performance was repeated 5 min later (T2) and once again without any rest (T3), in order to evaluate the warm-up phenomenon or paradoxical worsening. The time spent to perform the exercise was measured with a stopwatch. The test was performed when the patient was taking mexiletine, then 12 (test #1) and 21 (test #2) months after the introduction of flecainide. The test showed a remarkable improvement in both mother and son with flecainide treatment

A functional chair test [5] showed a remarkable improvement after flecainide introduction and a further improvement 9 months later (Fig. 1). The quality of life of the proband was evaluated with the 36-Item Short-Form Health Survey [6]. The physical component scale increased from 29 during mexiletine therapy to 38 after flecainide introduction. Improvement included physical functioning, role limitations due to physical problems, body pain, and general health perceptions. After almost 2 years of treatment no side effects have been reported.

There are only a few reports on myotonia permanens associated with G1306E mutation, including a familial case with autosomal dominant inheritance [7]. In this study, the proband did not have any symptom relief with mexiletine, oxcarbazepine, or thiazide diuretics. Although flecainide has been shown to be an effective hNav1.4 channel blocker in vitro [2, 8, 9], its clinical use as an antimyotonic agent is rarely reported [10]. The improvement of myotonia by flecainide obtained here suggests that there may be a relationship between the G1306E mutation and the response to flecainide therapy. Indeed we previously demonstrated that flecainide was more efficient than mexiletine in blocking functionally expressed G1306E channels, probably due to the positive shift of channel availability voltage-dependence by the mutation [2]. On the same basis, we may hypothesize that at least 15 out of 33 identified SCN4A mutations, which are known to induce a positive shift of voltage-dependence, may be more sensitive to flecainide than mexiletine, and patients carrying these mutations might have more benefit from flecainide. Such hypothesis merits further investigation.

The present study provides a paradigm of a bench-to-bedside pharmacogenetics strategy and indicates the relevance of pursuing the pharmacological characterization of sodium channel mutants in vitro in order to define a drug-genotype relationship. A clinical trial would be then warranted to develop a pharmacogenetic strategy to better address treatment in individual myotonic patients. Such an approach may be also of great interest in other familial diseases, including cardiac arrhythmias, epilepsies, and neuropathic pain, all characterized by mutations in sodium channel genes and by possible treatment with sodium channel blockers.
